# Reassessing the role of mitochondrial DNA mutations in autism spectrum disorder

**DOI:** 10.1186/1471-2350-12-50

**Published:** 2011-04-06

**Authors:** Vanesa Álvarez-Iglesias, Ana Mosquera-Miguel, Ivón Cuscó, Ángel Carracedo, Luis Alberto Pérez-Jurado, Antonio Salas

**Affiliations:** 1 Unidade de Xenética, Instituto de Medicina Legal and Departamento de Anatomía Patolóxica e Ciencias Forenses, Facultade de Medicina, Universidade de Santiago de Compostela, Galicia, Spain; 2 Unidad de Genética, Universitat Pompeu Fabra, Barcelona, Spain; 3 CIBER de enfermedades raras (CIBERER), Spain; 4 Fundación Pública Galega de Medicina Xenómica, SERGAS, Santiago de Compostela, Galicia, Spain; 5 Programa de Medicina Molecular y Genética, Hospital Universitari Vall d'Hebron, Barcelona, Spain; 6 Dept. of Genome Sciences, University of Washington, Seattle, WA, USA

## Abstract

**Background:**

There is increasing evidence that impairment of mitochondrial energy metabolism plays an important role in the pathophysiology of autism spectrum disorders (ASD; OMIM number: 209850). A significant proportion of ASD cases display biochemical alterations suggestive of mitochondrial dysfunction and several studies have reported that mutations in the mitochondrial DNA (mtDNA) molecule could be involved in the disease phenotype.

**Methods:**

We analysed a cohort of 148 patients with idiopathic ASD for a number of mutations proposed in the literature as pathogenic in ASD. We also carried out a case control association study for the most common European haplogroups (hgs) and their diagnostic single nucleotide polymorphisms (SNPs) by comparing cases with 753 healthy and ethnically matched controls.

**Results:**

We did not find statistical support for an association between mtDNA mutations or polymorphisms and ASD.

**Conclusions:**

Our results are compatible with the idea that mtDNA mutations are not a relevant cause of ASD and the frequent observation of concomitant mitochondrial dysfunction and ASD could be due to nuclear factors influencing mitochondrion functions or to a more complex interplay between the nucleus and the mitochondrion/mtDNA.

## Background

Autism/autistic spectrum disorders (ASD) are complex neurodevelopmental conditions affecting approximately one in 150 children. They are characterized by a disturbance in communication skills and reciprocal social interaction, along with restrictive and repetitive behaviours. There is increasing evidence that ASD have an important genetic component with aetiological heterogeneity, including association with several metabolic disorders [1,2]. Rare mutations in a few genes, copy number variants (CNVs) disrupting functional pathways and linkage or association to a number of different loci can account for the genetic aetiology or liability to ASD in up to 20% of cases [1,3-6].

Recent studies have also suggested that impairment of mitochondrial energy metabolism plays a role in the aetiology of ASD [7-9]. The study by Oliveira et al. [2] found that 7% of children in a population-based survey of school-age children with ASD met the criteria for mitochondrial respiratory chain disorders, and that they were also clinically indistinguishable from other children with ASD. This already recognized feature of ASD has lead several researchers to analyse mutations of the mtDNA as potential risk factors in ASD. Thus, Graf et al. [10] reported a family with a heterogeneous group of neurological disorders associated with the mtDNA 8363G > A transfer ribonucleic acid (RNA)^Lys ^mutation; the phenotype of one child in the family (heteroplasmic for this mutation) was consistent with ASD; however, there was not a solid reason to consider this mutation as responsible for ASD in that patient. Pons et al. [11] explored the association of mtDNA mutations with autistic spectrum disorders, with special emphasis on mutation A3243G and mtDNA depletion. These authors reported two ASD patients bearing the 3243A > G mutation located in the mtDNA tRNA^Leu(UUR) ^gene. This mutation typically causes mitochondrial encephalopathy with lactic acidosis and stroke-like episodes (MELAS) and has also been associated with developmental delay and seizures and maternally inherited progressive external ophthalmoplegia [11]. According to Pons et al. [11], ASD with or without additional neurological features can be an early presentation of the A3243G mutation and can be a prominent clinical manifestation of mtDNA depletion.

There are evidences suggesting that inherited mtDNA sequence variants also have a subtle influence on respiratory chain activity during a critical stage of neurodevelopment which is highly energy dependent; therefore, these variants could contribute to the complex aetiology of neurodevelopmental disorders such as ASD. Thus, with this hypothesis in mind, Weissman et al. [12] analyzed selected mtDNA mutations in 25 ASD patients with mitochondrial disorders. The authors detected the following mutations: 3397A > G and 4295A > G (both regarded as variants of probable pathogenicity), and 3394T > C, 10394C > T, 11809T > C and 11984T > C (regarded as variants of unclear pathogenicity) (see Table four in Weissman et al. [12]). According to the authors "*Each DNA sequence variant was evaluated for pathogenicity by a search of the MITOMAP, and mtDB-Human Mitochondrial Genome databases, PubMed, and compendia of mtDNA mutations including guidelines for determination of pathogenicity*". It is not clear, however, whether these mutations can be regarded as pathogenic. For instance, the scoring system proposed by Mitchell et al. [13] gives the 3397A > G mutation a low score (5 out of 40), while its 'pathogenic' status in MITOMAP is regarded as provisional. Phylogenetic considerations do not favour its disease status [14-16]: this variant appears independently in at least 11unrelated phylogenetic branches in apparently healthy individuals (see for instance http://www.genpat.uu.se/mtDB/).

Kent et al. [17] investigated the most common mtDNA variants defining the main European haplogroups hgs (clades of maternal lineages phylogenetically closely related) in ASD probands *versus *two healthy control groups. Their results showed no evidence of an association between mtDNA hg and ASD.

Therefore there is a certain amount of evidence that seems to suggest a role of mtDNA variants in ASD; however, this evidence is weak and/or has not been replicated yet in different independent cohorts of patients. The main aim of the present work is to explore the potential association of mtDNA mutations and well-known polymorphisms with both control and coding regions, and/or the hg background in patients ascertained for ASD.

## Methods

### Patients

The characteristics of a subset of the patients used in the present study are described in Cuscó et al. [18]. Briefly, a total of 148 Spanish patients (88 children followed in the neurology clinic and 60 institutionalized adults) with a confirmed diagnosis of one of the categories of ASD listed in the Diagnosis and Statistical Manual of Mental Diseases (DSM-IV) were included in the study. All patients were studied using the Autism Diagnostic Interview-Revised (ADI-R) instrument to define a specific category of ASD and the Wechsler Intelligence Scale for Children (WISC) or the Wechsler Adult Intelligence Scale (WAIS), allowing us to measure general, verbal and performance IQ, as well as analysis of multiple factorial components of cognitive functioning. The Leiter International Performance Scale-Revised (Leiter-R) and the Raven Progressive Matrices (RPM) were used for non-verbal patients. All patients had an extensive evaluation by neurologists and clinical geneticists along with an intensive laboratory workup including standard karyotyping, fragile-X molecular testing and subtelomeric and targeted Multiplex Ligation-Dependent Probe amplification (MLPA) assays (homemade panel designed to detect genomic duplications/deletions of specific regions associated with ASD and mental retardation: 1p36, 1q21.1, 2q37, 7q11.23, 15q11.2, 15q13.3, 16p11.2, 17p11.2, 22q11.2 and 22q13.3), molecular karyotyping by microarray comparative genomic hybridization in 2/3 of cases, as well as metabolic and brain imaging studies in cases where clinically indicated. Individuals with genetic or structural anomalies potentially causative of ASD were excluded from the study. Table 1 summarizes some of the medical and demographic features of the final 148 idiopathic ASD patients in the present study.

**Table 1:** Medical history and demographic data of ASD patients

	Adult cohort *n *= 60	Child cohort *n *= 88
**Sex (M/F ratio)**	2.3	4.5
**Age (Mean ± SD; years)**	42.8 ± 8	10.2 ± 4.2
**Ethnicity (%)**		
Western Europeans^1^	60 (100%)	81 (92%)
Other ethnicities	-	7 (8%)
**Epilepsy**	23 (38.3%)	9 (10.2%)
**Dysmorphism **^2^	22 (36.6%)	6 (6.8%)
**Mild mitochondrial dysfunction **^23^	1/9 (11%)	2/26 (7.7%)

^1^Western Europeans refers to people born mainly in Catalonia (northeast Spain), but some are from other Spanish locations;

^2 ^All patients were examined by at least two clinical geneticists. Body and facial measurements were compared with normal ranges for age. Microcephaly or macrocephaly were defined by measures of the occipitofrontal head circumference (OFC) below the 3^rd ^centile or above 97^th ^centile, respectively. In this study we classified the patients as "dysmorphic" if they had three minor anomalies or more, abnormal growth or OFC parameters, or at least one major malformation.

^3 ^Slight elevation of the lactic acid in blood or urine in single or repeated sampling

### Control group

Two different control group samples were used in the present study. A control group of DNA samples from 137 (population control) individuals matched for population ancestry (Spanish anonymous blood donors) were collected and analysed for the presumed pathogenic mutations. The second control group, referred as CG2, as used in Salas et al. [19], consisted of 616 healthy Spanish individuals. The two control groups were combined (*n *= 753) for the case-control association study involving the polymorphisms which define the main European hgs.

### DNA extraction

Genomic DNA was extracted from blood samples using a salting-out method and employing the Puregene DNA Purification Kit (Gentra Systems; Minneapolis; USA).

### Ethical approval

All subjects participated after written informed consent was obtained from their families or other legal caregivers. The DNA extracts were submitted to the laboratory in Santiago de Compostela for genotyping. In addition, this study was approved by the Ethical committee of the University of Santiago de Compostela and conformed to the Spanish Law for Biomedical Research (Law 14/2007- 3^rd ^July).

### Automatic sequencing

The mtDNA hypervariable region I (HVS-I) segment was sequenced in forward and reverse directions and were previously reported in Álvarez-Iglesias et al. [20].

### Genotyping of mtDNA variants

The following 25 well-known pathogenic mutations in mitochondrial disorders were genotyped in all of the patients: 3243A > G, 3460G > A, 3697G > A, 3946G > A, 3949T > C, 7445A > G, 7445A > C, 8993T > G, 8993T > C, 9176T > C, 9176T > G, 10158T > C, 10191T > C, 10663T > C, 11777C > A, 11778G > A, 11832G > A, 12706T > C, 13513G > A, 13514A > G, 14459G > A, 14482C > A, 14482C > G, 14484T > C, and 14487T > C. Genotyping was carried out using the minisequencing assay reported in Álvarez-Iglesias et al. [21].

In addition, the six mtDNA mutations reported by Weissman et al. [12] as being probable (3397A > G, 4295A > G) or unclear (3394T > C, 10394C > T, 11809T > C and 11984T > C) pathogenic mutations were also genotyped (see their Table four). A new minisequencing assay was designed *ad hoc*, following the methodology reported before [21,22].

The hg status of several mtDNA profiles cannot be inferred solely from control region data. We therefore genotyped the mtSNPs defining the main branches of the European mtDNA phylogeny, as done in the study by Quintáns et al. [23]. These polymorphisms were used for a case-control association study.

### Association study

A case-control association study was carried out between ASD patients (*n *= 148) and controls (*n *= 753) for hg status, and individually for each mtSNP. Firstly, allele frequencies between cases and controls were compared in order to assess individual mtSNP associations using a one degree of freedom chi-square test (or Fisher's exact test for cell counts below five). A nominal value of α = 0.05 was selected to assess the significance of the association. Secondly, these same statistical tests were used to carry out association analyses for hgs in cases and controls by comparing the frequency of each hg versus the other hgs aggregated. A permutation test was employed to address the issue of multiple testing (see footnote of Table 2).

**Table 2:** Pearson's chi-square test for cases versus controls of SNPs and most frequent hgs

	**rCRS ref**.^**a**^	**MA**^**b**^	**MAF**_**CA**_^**c**^	**MAF**_**CO**_^**c**^	CHI2 Exact	*P*-value	**Adjusted *P-*value**^**d**^	**OR (95% CI)**^**e**^
**3010G > A**	G	A	0.24	0.27	0.557	0.455	1	0.85 (0.56-1.29)
**3915G > A**	G	A	0.02	0.04	0.874	0.350	0.995	0.57 (0.17-1.89)
**3992C > T**	C	T	0.00	0.01	1.320	0.251	0.979	-
**4216T > C**	T	C	0.13	0.16	0.939	0.333	0.993	0.77 (0.46-1-30)
**4336T > C**	T	C	0.04	0.04	0.047	0.828	1	1.11 (0.45-2.72)
**4529A > T**	A	T	0.01	0.01	0.182	0.669	1	0.64 (0.08-5.13)
**4580G > A**	G	A	0.06	0.03	1.620	0.203	0.941	1.69 (0.75-3.82)
**4769A > G**	A	A	0.02	0.02	0.060	0.799	1	1.17 (0.34-4.04)
**4793A > C**	A	G	0.00	0.01	1.166	0.280	0.987	-
**6776T > C**	T	C	0.04	0.05	0.577	0.448	1	0.69 (0.27-1.79)
**7028C > T**	C	C	0.40	0.44	0.683	0.409	0.999	1.16 (0.81-1.67)
**10398A > G**	A	G	0.15	0.20	2.010	0.156	0.902	0.70 (0.43-1.15)
**10400C > T**	C	T	0.02	0.01	1.023	0.312	0.991	1.97 (0.52-7.52)
**10463T > C**	T	C	0.09	0.09	0.001	0.982	1	1.01 (0.53-1.92)
**10873T > C**	T	C	0.06	0.04	1.609	0.205	0.951	0.61 (0.28-1.32)
**12308A > G**	A	G	0.19	0.23	1.270	0.260	0.982	0.77 (0.49-1.21)
**12705C > T**	C	T	0.14	0.08	4.857	0.028	0.315	1.82 (1.06-3.13)
**13966A > G**	A	G	0.02	0.02	0.001	0.972	1	1.02 (0.29-3.58)
**14766C > T**	C	T	0.47	0.49	0.242	0.622	1	0.91 (0.64-1.31)
**H**	-	-	0.40	0.44	0.791	0.374	0.999	0.85 (0.59-1.22)
**H1**	-	-	0.16	0.19	0.689	0.406	0.999	1.22 (0.76-1.96)
**HV**	-	-	0.52	0.50	0.121	0.728	1	1.06 (0.75-1.51)
**U**	-	-	0.19	0.23	1.248	0.264	0.983	1.29 (0.83-2.01)
**J**	-	-	0.03	0.08	4.411	0.036	0.390	2.61 (1.03-6.61)
**T**	-	-	0.09	0.08	0.077	0.782	1	0.92 (0.49-1.71)
**JT**	-	-	0.12	0.16	1.727	0.189	0.941	1.42 (0.84-2.41)

rCRS = revised Cambridge Reference Sequence; MA = minor allele; MAF = minimum allele frequency.

^a^rCRS: allele in the revised Cambridge Reference Sequence (rCRS)[41];

^b^MA: minor allele;

^c^MAF: minimum allele frequency computed on cases (MAF_CA_) and control (MAF_CO_) individuals;

^d^Adjusted *P*-value: adjustment of chi-square *P*-values was carried out with a permutation-based approach; number of permutations = 20,000;

^e^OR (95%CI): ORs were computed with the rCRS allele as a reference

The statistical analyses were carried out using the statistical packages Stata v.8 (http://www.stata.com/) and R (http://www.r-project.org/).

Quanto software [24] was used for power calculations. See Salas et al. [19] for some caveats.

## Results

### Analysis of mtDNA mutations in ASD patients

Additional file 1: Table S1 shows the mtDNA control region sequences and the minisequencing results of patients and a control group which consist of 137 healthy individuals (see Materials and methods). All of these individuals and a second Spanish control group consisting of 616 people were genotyped for a set of mtSNPs diagnostic of the main mtDNA European hgs. All of this information put together indicated that the patients were very representative of the frequency pattern of a typical Spanish population [20,25]. Haplogroup H was the most common in the cases (40%) and controls (44%), and H1 was its most prevalent sub-lineage (16% in the cases and 19% in the controls). Other well-known hgs were present, such as U (where K is nested phylogenetically), J, T, V and W. However, a few non-European lineages were observed in our patients. Although there were not coding region SNPs to assist hg classification, some of these non-European mtDNAs can be clearly allocated to typical East Asian lineages. However, given the fact that immigration from South America to Spanish cosmopolitan cities (such as Barcelona) significantly increased during the last decade, it is highly probably that these Asian lineages belonged to Native American branches of the Asian phylogeny, i.e. hg A2, B2, C1 and D1 [26-28]. In addition, a few individuals undoubtedly carried typical sub-Saharan lineages belonging to different L-hgs (Additional file 1: Table S1)[29,30].

In addition, a total of 25 well-known pathogenic mtDNA mutations were minisequenced in all patients and 137 controls. All patients were carriers of non-pathogenic wild-type variants. We also genotyped the six mutations reported in Weissman et al. [12] as being of probable pathogenicity or of uncertain pathogenic significance; only 3397A > G was observed in one patient (AU010), while 3394T > C was identified in two healthy controls. It is important to note that AU010 did not suffer any mitochondrial disorder. Given the fact that the pathogenic status of these variants is debatable, it was not surprising to detect similar frequencies in cases and controls.

### Case-control association study

A case-control association study was carried out for 148 patients and 753 controls. The mtSNPs genotyped represent well-known branches of the West European mtDNA phylogeny. Pearson's chi-square or Fisher's exact tests were used to assess mtSNP associations. The best *P*-value (Pearson's χ2 test, nominal *P*-value = 0.028) was found for mtSNP 12705C > T, which lead from macro-hg N to R; this mtSNP apparently increases the risk of suffering from ASD as indicated by the OR value (OR = 1.82; 95% CI = 1.06-3.13); however, this significance was not maintained after correcting for multiple hypotheses using a permutation test procedure (adjusted *P*-value = 0.315) (Table 2), as done in previous studies[19,31].

The association test was only carried out on the most frequent hgs in order to increase the probability of detecting any association. The frequency of mtSNPs and hgs in Iberia is already known from empirical population studies [20]. The *a priori *power to detect odds ratios as low as two for mtSNPs and hgs with frequencies higher than 20% was about 96%; whereas a statistical power of 80% and OR = 1.5 can only be obtained for the most frequent mtSNPs or hgs.

## Discussion

The brain is strongly dependent on the ATP production of the cell energy-producing organelle, the mitochondrion. Therefore, adequate mitochondrial metabolism is essential for normal brain functions. There is a large body of evidence involving mitochondrial dysfunctions in ASD [6,32,33]. While some evidence points to candidate nuclear DNA (nDNA) loci influencing mitochondrial functions [2,34,35], other evidence seems to imply that mtDNA mutations are potential causes of ASD [11]. Although the implication of nDNA factors has found relatively strong support, the presumable role of the mtDNA genome is still weak. The present study aimed to provide more evidence to support the role of mtDNA mutations or polymorphisms in ASD by screening one of the largest cohort of patients analysed to date. We tried to screen the mtDNA of these patients for a wide spectrum of mutations and polymorphisms in order to explore the presumable roles of high penetrance mtDNA mutations or low penetrance common polymorphisms in ASD.

We targeted 25 well-known high penetrance pathogenic mutations in the mtDNA genome. All of these mutations are commonly responsible for a wide spectrum of mitochondrion diseases. We did not observe any of these mutations, either in homoplasmic or heteroplasmic conditions, in the patients or controls analysed in the present study. It is therefore unlikely that well-known pathogenic mutations are responsible for a significant proportion of ASD.

In addition, a set of six other mutations regarded as unclear or probable pathogenic by Weissman et al. [12] were also targeted in our patients. We did not observe any of these mutations in our patients or in a sample of healthy controls, with only anecdotic exceptions. As inferred from the complete genome sequences available in GenBank and the literature, several of these mutations are likely to be rare variants that normally characterize mtDNA genomes in populations or even in some minor hgs (e.g. 10394C > T is the unique diagnostic site for H16, [20,36]). The fact that these variants are non-synonymous or highly conserved in comparisons among species does not guarantee their disease status [19]. Many of these variations are normally located at the tips of the phylogenies in the mtDNA tree (see e.g. http://www.phylotree.org), although their rarity is often interpreted as indicative of their pathogenic condition by many authors without any solid evidence [14,15,37]. The mere appearance of a mutation in MITOMAP reporting it as pathogenic has been over-interpreted [16]. The results of the present article indicate that it is unlikely that these mutations play an important role in ASD.

We also carried out a case-control association study in ASD patients and we could not find any evidence relating common polymorphisms to ASD. Although we did not detect a statistical association between cases and controls, it is important to be aware of the presence of non-European mtDNA lineages in cohorts of patients and controls because these haplotypes could provide the basis for a false positive finding of association (due to population stratification). Therefore, the mtDNA hg background as analysed in the present study does not seem to be a risk or protection factor in ASD. A type II error due to a lack of statistical power cannot be fully rule out, though this possibility is more unlikely for the most frequent mtSNPs and hgs. Analysis of entire mtDNA genomes could also provide new insights about the potential role (if any) of other rare variants; however, very large sample sizes would be necessary to achieve an suitable statistical power. Next-Generation Sequencing (NGS) could perhaps bring the opportunity for large cohort of samples to be analyzed for entire mtDNA genomes; unfortunately, the few recent attempts of NGS mtDNA genomes do not seem to hold the necessary quality standards (Bandelt and Salas: Current Next Generation Sequencing technology may not meet forensic standards, submitted) when evaluated under a phylogenetic perspective [38,39]. The role of mtDNA gene dosage in ASD as very recently assessed by Giulivi et al. [33] would also be a good target for future replication. Therefore, although previous findings are in agreement with our results [17], we consider that further studies are needed in order to definitely rule out any role of mtDNA hgs in ASD.

As reviewed by Palmieri and Persico [32], regarding ASD, oxidative phosphorylation (OXPHOS) in the mitochondrion requires at least 80 proteins, of which only 13 are encoded by the mtDNA, while mitochondrial functioning has been estimated to need the participation of approximately 1500 nuclear genes [40]. Any of these nuclear genes or copy number variations (CNVs) could explain the mitochondrial defects observed in a small minority of ASD patients [40].

## Conclusions

Although it is widely accepted that some forms of ASD appear concomitantly with the impairment of mitochondrial energy metabolism, there are reasons to believe that the cause of these mitochondrial disorders does not systematically rest on mutations or variants in the mtDNA molecule. Pathogenic mtDNA mutations have been reported in ASD patients, but this seems to be the exception rather than the rule. It is more likely that the real causes of mitochondrial deficiencies in some ASD cases are due to the intervention of several nuclear factors acting alone (additively or epistatically) or through a complex interplay with mtDNA variants. For the time being, while the cause for mitochondrion dysfunction in ASD remains unclear, there is no reason to indicate systematic screening for mtDNA mutations in ASD patients unless a mitochondrion disorder is suggested by a clear phenotype.

## Competing interests

The authors declare that they have no competing interests.

## Authors' contributions

IC and LAPJ provided patient and control samples and participated together with VAI and AS to the design and coordination of the study. AC, LAPJ, and AS contributed laboratory materials and reagents. VAI and AMM carried out the molecular genetic analysis; while VAI and AS carried out the phylogenetic and phylogeographic analyses. AS drafted the manuscript and performed the statistical analyses; and all the other authors contributed to the writing. All of the authors critically read the manuscript and approved the final manuscript.

## Pre-publication history

The pre-publication history for this paper can be accessed here:

http://www.biomedcentral.com/1471-2350/12/50/prepub

## Supplementary Material

Additional file 1 **Table S1**. MtDNA control sequences and coding region mtSNP genotypes. Mutations are referred to with respect to the rCRS. Transitions are omitted while transversions are indicated as a suffix. A "+" indicates an insertion whereas "del" refers to a deletion. See text for hg assignation criteria. Click here for file
